# Comparative Evaluation of Luminex xTAG^®^ Gastrointestinal Pathogen Panel and Direct-From-Stool Real-Time PCR for Detection of *C. difficile* Toxin *tcdB* in Stool Samples from a Pediatric Population

**DOI:** 10.3390/microorganisms10112214

**Published:** 2022-11-09

**Authors:** Hannah Tyrrell, Sarah B. N. Morin, Colin D. Lloyd, Brendon Parsons, Taryn Stokowski, Jianling Xie, Ran Zhuo, Bonita E. Lee, Xiao-Li Pang, Stephen B. Freedman, Linda Chui

**Affiliations:** 1 Department of Laboratory Medicine and Pathology, University of Alberta, Edmonton, AB T6G 1C9, Canada; 2 Department of Pediatrics, Cumming School of Medicine, University of Calgary, Calgary, AB T3B 6A8, Canada; 3 Department of Pediatrics, Women and Children’s Health Research Institute, University of Alberta, Edmonton, AB T6G 1C9, Canada; 4 Alberta Precision Laboratories: Public Health Laboratory, Edmonton, AB T6G 2J2, Canada; 5 Sections of Pediatric Emergency Medicine and Gastroenterology, Departments of Pediatrics and Emergency Medicine, Alberta Children’s Hospital Research Institute, Alberta Children’s Hospital, Cumming School of Medicine, University of Calgary, Calgary, AB T3B 6A8, Canada

**Keywords:** Luminex xTAG^®^ GPP, real-time PCR, stool, *C. difficile*, children, culture

## Abstract

Detection of *Clostridioides difficile* toxins in patients with gastroenteritis has increasingly been accomplished through the use of enteric multiplex syndromic panels. Comparisons of the performance of these panels to both direct-from-stool (DFS) and culture-enriched stools followed by polymerase chain reaction (PCR) methods in pediatric populations are limited. Here, we compare the performance of the Luminex xTAG^®^ Gastrointestinal Pathogen Panel (GPP) to our DFS in-house real-time PCR (DFS RT-PCR) assay for the detection of *C. difficile* toxin gene, *tcdB*, using 2641 stool specimens collected from children enrolled in the Alberta Provincial Pediatric EnTeric Infection Team (APPETITE) study in Alberta, Canada. We used culture enrichment followed by in-house RT-PCR to resolve discordant results between the two assays. We found excellent agreement (*k* = 0.89) between the GPP and our DFS RT-PCR assay: the positive percent agreement between the two assays was 97%, and the negative percent agreement was 99%. GPP, a multi-analyte platform can easily be implemented into a routine diagnostic laboratory for detecting enteric pathogens including *C. difficile*.

## 1. Introduction

*Clostridioides difficile* is an anaerobic, spore-forming, toxin-producing, Gram-positive bacillus bacterium. Infection with *C. difficile* can result in gastrointestinal symptoms, including diarrhea, pseudomembranous colitis, and toxic megacolon [1]. These symptoms are largely due to the presence of *C. difficile* virulence factors such as toxin A (TcdA), toxin B (TcdB), and/or the binary toxin (CDT) [1,2]. The virulence genes, *tcdA* and *tcdB* are encoded by the pathogenicity locus, while the *cdt* genes are encoded by the binary toxin locus. Strains that lack the toxin genes are considered non-pathogenic [1].

Historically, cell culture cytotoxicity neutralization assays have been used for the detection of *C. difficile*. However, this method is labour-intensive, has a long turnaround time, and is not cost-efficient. Currently, the majority of diagnostic laboratories have switched to enzyme immunoassay (EIA) for the detection of *C. difficile* toxins [3], or nucleic acid amplification tests (NAAT) with DNA extracted directly from stool samples which offer greater detection sensitivity [3,4,5,6,7]. Automated multiplex syndromic panels utilizing polymerase chain reaction (PCR) to detect multiple pathogens simultaneously within a few hours [6,7,8] have been implemented in the majority of high-volume frontline diagnostic laboratories. Multiplex panels have been evaluated for their detection of *C. difficile* toxin gene targets [8,9,10,11,12,13,14,15], but primarily in adult cohorts [8,12,13,16].

In this study, we evaluated the diagnostic characteristics of an in-house direct-from-stool real-time PCR (DFS RT-PCR) assay for *C. difficile tcdB* detection and compared performance between the DFS RT-PCR and the Luminex xTAG^®^ Gastrointestinal Pathogen Panel (GPP) (Luminex Corporation, Toronto, ON, Canada), a multiplex gastrointestinal syndromic panel, for the detection of *tcdB* using stool samples previously enrolled in the Alberta Provincial Pediatric EnTeric Infection Team (APPETITE) study in Alberta, Canada [17]. Additionally, we employed an enrichment culture approach to further characterize the differences observed between *tcdB* detection by DFS RT-PCR and GPP.

## 2. Materials and Methods

### 2.1. Clinical Samples

A total of 2641 stool samples included in this study were collected from children (≤17.99 years old) enrolled in the Alberta Provincial Pediatric EnTeric Infection TEam (APPETITE) study from two major cities in Alberta, Canada and were stored at −70 °C [17]. This study was approved by the ethics boards of the University of Alberta (Approval code: Pro00050790, Approval Date: 2014) and the University of Calgary (Approval code: REB14-1112, Approval Date: 2014).

### 2.2. Nucleic Acid Extraction

Aliquots of liquid stool (100 μL) or solid stool (100–150 mg) were suspended in 1 mL of NucleiSENS^®^ Lysis Buffer (bioMérieux, Montreal, QC, Canada) in a Bertin Corp SK38 Soil grinding lysis bead tube (ESBE Scientific, Saint-Laurent, QC, Canada), and vortexed for ten minutes. Samples were then incubated at room temperature for 15 min and then centrifuged at 15,871× *g* for five minutes. Nucleic acid was then extracted from 200 μL of lysate via NucleiSENS^®^ easyMAG^®^ instrument (bioMérieux, Montreal, QC, Canada). Following extraction, 70 μL of the eluate was collected and stored at −70 °C [18].

### 2.3. Molecular Testing

The Luminex xTAG^®^ GPP is a multi-analyte platform targeting 15 enteric pathogens: *Shigella* spp., *Salmonella* spp., *Yersinia enterocolitica*, *Campylobacter* spp., *Vibrio cholerae,* Shiga-toxin producing *Escherichia coli*, enterotoxigenic *E. coli*, *E. coli* O157, adenovirus 40/41, rotavirus A, norovirus GI/GII, Giardia lamblia, Entamoeba histolytica, *Cryptosporidium* spp., and *C. difficile* toxin A/B [8]. Nucleic acid extracts of all stool samples (*n* = 2641) were tested using the GPP as per the manufacturer’s instructions.

Based on the results of our previous investigation in the APPETITE study (data not shown), the samples tested by GPP were either *tcdA* and *tcdB* positive or only *tcdB* positive. Consequently, only the target for *tcdB* was used in this study for DFS RT PCR on the same nucleic acid extracts prepared for the GPP assay. Five μL of extracted nucleic acid was used as template in the PCR assay in a total volume of 25 μL. The primers and probe for *tcdB* were developed by Kubota et al. [19] (Table 1). Singleplex RT-PCR assays for *tcdB* were performed using the Applied Biosystems^™^ 7500 Fast instrument (Thermo Fisher Scientific, Waltham, MA, USA) with 5 μL template DNA, 222 nM forward and reverse primers, 333 nM probe, and 1× PrimeTime^®^ Gene Expression Master Mix (Integrated DNA Technologies, Skokie, IL, USA) in a final volume of 25 μL, and with the following amplification conditions: 1 cycle (95 °C × 20 s), 40 cycles (95 °C × 3 s, 60 °C × 30 s). DNA extracted from a toxigenic *C. difficile* strain was used as positive control for *tcdB* detection in addition to a no-template control for all RT-PCR assays.

### 2.4. Assessment of In-House tcdB RT-PCR

To determine the analytical specificity of the *tcdB* DFS RT-PCR, a panel of bacteria (Supplementary Table S1) was obtained from the Alberta Precision Laboratories-Provincial Laboratory for Public Health (ProvLab) Quality Control Department (Edmonton, AB, Canada). These organisms were grown in their respective culture media under appropriate growth conditions. Nucleic acid was extracted by suspending a single colony of bacteria in 100 μL rapid lysis buffer (100 mM NaCl, 10 mM Tris-HCl pH 8.3, 1 mM EDTA pH 9.0, 1% Triton X-100) and incubated for 15 min at 95 °C using a VWR Standard Heatblock (VWR Scientific, Mississauga, ON, Canada). Following incubation, the cell suspension was subjected to centrifugation of 13,000× *g* for 15 min and the supernatant containing extracted nucleic acid was removed and stored at −70 °C until used.

The analytical sensitivity of the RT-PCR was determined using serial dilutions of a toxigenic *C. difficile* strain isolated from a clincal specimen. The isolate was grown on Brain Heart Infusion (BHI) agar (ProvLab, Edmonton, AB, Canada) and a sweep of the culture was added to BHI broth and incubated for 6 h at 35 °C. The broth was then diluted to an optical density of 0.4, and ten-fold serial dilutions from 10^−1^ to 10^−8^ were prepared. Aliquots of 100 μL from dilutions 10^−5^ and 10^−6^ were inoculated onto BHI agar (ProvLab, Edmonton, AB, Canada) in triplicates and incubated for 48 h in anaerobic conditions to numerate the colony forming units/mL (CFU/mL), and RT-PCR was performed for all dilutions of cell suspensions using nucleic acids extracted by the NucleiSENS^®^ easyMAG^®^ instrument (bioMérieux) as outlined in Section 2.2, with the modification of using 200 µL of cell suspension in 800 µL of NucleiSENS^®^ Lysis Buffer (bioMérieux). Both the bacterial culture, to determine the CFU/mL, and the RT-PCR assays (both performed in triplicates) were repeated in 10 different experiments on separate days to establish the reproducibility of the assay. The limit of detection was determined based on the results of the sensitivity assay as described above. The positive cut-off for the RT-PCR assay was set to the Ct value obtained where a positive amplification curve was seen in 95% of the PCR runs in 10 replicates (Supplementary Table S2).

### 2.5. Preparation for Stool Specimens for Culture Enrichment

**Figure 1:**
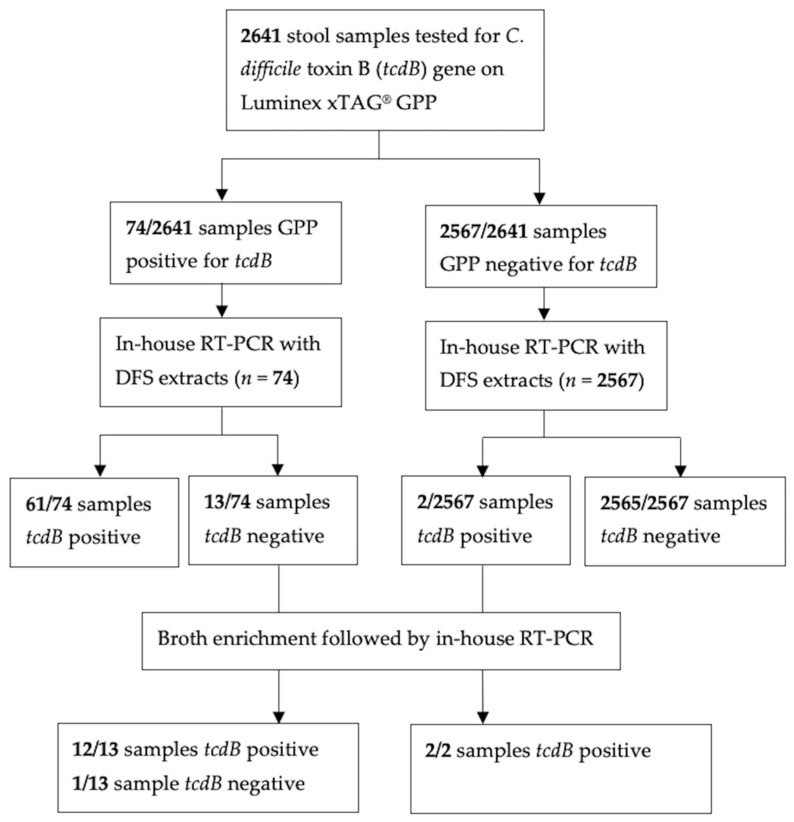
Flow diagram of testing algorithm using the GPP, DFS RT-PCR, and PE RT-PCR. Abbreviations: GPP, Luminex xTAG^®^ Gastrointestinal Pathogen Panel; DFS, Direct-from-stool; PE, Post enrichment.

Enrichment of *C. difficile* by culture was only performed for stool samples with discordant GPP and in-house RT-PCR results (Figure 1). Stool samples were prepared by suspending 250 μL liquid stool in 250 μL 1× phosphate-buffered saline (PBS) or ~100 mg solid stool in 500 μL 1× PBS. Alcohol shock and selective media were used to improve *C. difficile* culture in the stool. A 500 μL of 95% ethanol was added to the stool suspension. The mixture was vortexed until homogenized, incubated at room temperature for 35 min and centrifuged at 1150× *g* for 5 min. The pellet was swabbed using a sterile cotton swab and inoculated into Hardy Diagnostics C Diff Banana Broth™ (Micronostyx, Ottawa, ON, Canada), and incubated at 35 °C for 24 to 72 h. For broths that underwent a colour change from yellow to red (indicative of bacterial growth) after enrichment, 100 μL of broth was added to 1 mL NucleiSENS^®^ Lysis Buffer (bioMérieux) in a Bertin Corp SK38 soil grinding lysis bead tube for nucleic acid extraction as described in Section 2.2. It can be assumed that samples which tested positive by both assays would test positive using in-house RT-PCR after *C. difficile* broth post enrichment (PE), and samples which tested negative by both assays would test negative by PE RT-PCR. Consequently, the diagnostic sensitivity and specificity of GPP and DFS in-house RT-PCR were calculated using the discordant sample results obtained by PE RT-PCR (i.e., using this method to resolve results as either true positives or true negatives) [20].

### 2.6. Statistical Analysis

The significance of the difference in the results of the same samples tested by both GPP and DFS in-house RT-PCR was assessed using McNemar analysis (α = 0.05). Comparison of the two assays was summarized using two parameters: (1) positive percent agreement (PPA) calculated as 100% × [(*a*)/(*a* + *c*)], where *a* = the number of samples tested positive by both assays and *c* = the number of samples that tested positive by DFS in-house RT-PCR and negative by GPP, and (2) negative percent agreement (NPA), calculated as 100% × [(*b*)/(*b* + *d*)], where *b* = the number of samples that tested negative by both assays, and *d* = the number of samples tested negative by DFS in-house RT-PCR and positive by GPP [20]. The overall agreement of GPP and the in-house RT-PCR were analyzed with Cohen’s Kappa. We defined kappa scores of 0.6 to 0.69 as moderate, 0.7 to 0.8 as good and above 0.8 as excellent agreement [21]. The Ct values of the DFS RT-PCR of the samples tested positive for *C. difficile* between participants with acute gastroenteritis and those with no gastroenteritis, and between participants who were <2 years and those ≥2 years, as *C. difficile* more often represents colonization (i.e., not infection) in young children [22] were compared using Mann–Whitney U test. Fisher’s exact test was used to evaluate whether the frequency of concordance varied by participant age using 2 years as the cut-off. Statistical significance was set at *p* < 0.05 and analyses were performed using RStudio (Version 1.3.1073, Boston, MA, USA).

## 3. Results

In the specificity panel for *tcdB* RT-PCR assay only *C. difficile* was positive for the *tcdB* gene and no cross reactivity was observed with the other organisms in the specificity panel (Supplementary Table S1). The results from the sensitivity panel indicated that the limit of detection is 10^4^ colony forming units (CFU)/mL and we established the Ct cutoff value of our assay at 35 (Supplementary Table S2).

GPP testing and DFS RT-PCR were performed on 2641 clinical stool samples. Of the 2641 stool samples tested by GPP, 74 (2.8%) toxin-positive *C. difficile* samples were detected based on the presence of the *tcdB* target. Sixty-one (2.3%) samples were both GPP positive and DFS RT-PCR positive for *tcdB,* while 13 (0.5%) samples were GPP positive for *tcdB* but were negative by DFS RT-PCR. Conversely, 2565 samples (97.2%) were both GPP negative and DFS RT-PCR negative for *tcdB*, and two samples (0.1%) were GPP negative for *tcdB* but were tested positive for *tcdB* by DFS RT-PCR (Figure 1). In total, fifteen (0.6%) of the samples tested yielded discordant results between the GPP and DFS RT-PCR. Discordance was more likely to be GPP positive/DFS RT-PCR negative than GPP negative/DFS RT-PCR positive.

The agreement between DFS RT-PCR and GPP was excellent [k = 0.89, 95%CI (0.83, 0.94)]. The PPA between DFS RT-PCR and GPP was 96.8% [95% CI (89.0%, 99.6%)], while the NPA between DFS RT-PCR and GPP was 99.5% [95% CI (99.1%, 99.7%)]. McNemar analysis comparing the results of the GPP and DFS RT-PCR demonstrated that the difference between the two tests was significant (*p* = 0.0098).

With the addition of a culture enrichment and repeat RT-PCR, twelve of the thirteen (92.3%) GPP positive/DFS RT-PCR negative discordant samples were resolved after enrichment by culture, while one sample (7.7%) remained negative (Table 2). Both of the discordant GPP negative/DFS RT-PCR positive samples tested positive following culture enrichment (Table 2 and Figure 1). After the discordant samples were resolved using culture-enriched RT-PCR, we determined that the analytical sensitivity and specificity of GPP was 97.3% (95% CI [90.7%, 99.7%] and 100% (95% CI [99.8%, 100%] and of DFS RT-PCR was 84.0% (95% CI [73.7%, 91.4%]) and 100% (95% CI [99.9%, 100%], respectively.

**Figure 2:**
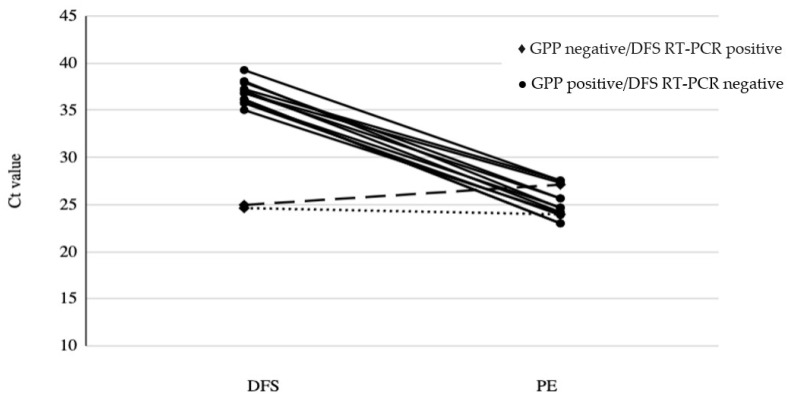
Comparison of Ct values for *tcdB* amplification from DFS and PE of the same samples.

Overall, PE RT-PCR Ct values among the discordant samples were improved as compared to the DFS RT-PCR Ct values before culture enrichment (Figure 2, Supplementary Table S3).

Only discordant samples that produced Ct values in both DFS RT-PCR and PE RT-PCR were included (*n* = 13). The cut off Ct values of the RT-PCR assay is at 35. All samples Ct values showed an average decrease of 12 cycles following PE RT-PCR except sample 2^♦^ (dashed line) and sample 9^♦^ (dotted line), which showed minimal changes in the Ct values. These 2 samples were tested negative for GPP assay but positive for DFS RT-PCR before culture enrichment.

There was no significant difference in terms of Ct values of DFS RT-PCR between 40 participants with acute gastroenteritis (median 27.6, interquartile range: 26.6–30.9) and the 22 without gastroenteritis (median 28.3, interquartile range: 25.2–31.6). The Ct values of DFS RT-PCR of the participants <2 years were significantly lower than those ≥2 years, (median 27.6 interquartile range: 26.2–30.9 and median 32.6, interquartile range: 31.8–33.4, respectively, *p* < 0.001). The frequency of concordance samples did not differ significantly between children age <2 and ≥2 years [99.3% (1597/1607) vs. 99.7% (1029/1032); *p* = 0.18].

## 4. Discussion

Our in-house RT-PCR assay targeting the *tcdB* gene has a sensitivity of 10^4^ CFU/mL (Supplementary Table S2) and showed no cross reactivity with any of the organisms included in the specificity panel (Supplementary Table S1). The correlation between DFS RT-PCR and GPP was excellent [k = 0.89, 95%CI (0.83, 0.94)]. By using DFS RT-PCR for comparison with GPP results, we found high positive and high negative agreement between the two tests for toxigenic *C. difficile* detection. Our findings of a PPA of 97% and NPA of 99% align with previous studies comparing percent agreements for detection of *C. difficile* toxins when conventional microbiological methods were used as reference standards for comparison with the GPP [23].

Despite the high agreements between the two assays, there was a significant difference in the results obtained by DFS RT-PCR and the GPP. Discordance between the tests was primarily observed in samples that tested GPP positive but DFS RT-PCR negative. Overall, 15 discordant samples (Figure 1) were identified, and these were alcohol shocked and enriched in C Diff Banana Broth™ to determine if the discordance observed was potentially due to differences in bacterial load. The results generated from the extracted DNA as template for PE RT-PCR showed an average difference of 12 cycles with lower Ct value using PE RT-PCR, suggesting an increase in target concentration of almost 4000-fold (Figure 2 and Supplementary Table S3). These findings supported that the alcohol shock followed by broth enrichment enhanced the culture of *C. difficile* [24,25] resulting in the decrease of Ct values in the RT-PCR assay as compared with using stool samples directly (Figure 2). However, two samples did not follow the overall trend. The Ct value of Sample 9 tested using PE RT-PCR was similar to the Ct value of DFS RT-PCR after the alcohol shock enrichment treatment, whereas, for Sample 2, the PE RT-PCR Ct value was higher than the DFS RT-PCR by 2 (Ct 24.96 to Ct 27.11) (Figure 2 and Supplementary Table S3). This indicated that there was likely minimal effect of the alcohol shock enrichment on these two stool samples. Interestingly, both Sample 2 and Sample 9 tested negative by GPP and positive by DFS RT-PCR. These results further support the assumption that both results are true positive even though there were very minor differences in the RT-PCR results with direct-from-stool and enriched culture post alcohol shock treatment. As for Sample 15 which was GPP positive/DFS RT-PCR negative, it still tested negative by PE RT-PCR suggesting that it was a false positive by the GPP assay. Alternatively, this discordance between the GPP and DFS RT-PCR could be the result of sampling error used for the shock enrichment or contamination during the GPP run [26]. Interestingly, the rate of false positivity of GPP we detected in our study (1/2641) was much lower than that of the false negative results generated by DFS RT-PCR (12/2641) suggesting that false positivity is less of an issue for the GPP assay targeting *C. difficile*.

In this study, nucleic acid degradation during storage between testing by the GPP and DFS RT-PCR assays might have contributed to the discordance between the two assays. All the nucleic acid extracts were stored at −70 °C but unfortunately, they were not aliquoted as “one-time-use” samples and were subjected to freeze–thawed cycles for different testing. The freeze–thawed cycles may have affected the integrity of the nucleic acid and resulted in false negatives in the RT-PCR results. Based on the level of detection of the RT-PCR, the cutoff Ct value of the assay is at 35, and all the discordant samples except for samples 2 and 9, are considered as DFS RT-PCR negative/indeterminate as shown in Supplementary Table S3. However, the sensitivity of the in-house RT-PCR assay did improve after the alcohol shock-enrichment treatments.

Interestingly, there was no difference in Ct values of DFS RT-PCR between participants who had acute gastroenteritis as compared to control patients with no gastroenteritis. On the other hand, children < 2 years, more likely to be only colonized with *C difficile*, had lower Ct values suggesting that the copies of toxin genes in their stool samples were higher than older children who would more likely be infected with *C difficile*. However, only 2 children in our study were ≥2 years; more studies with a larger sample size are needed to clarify this point. The frequency of concordance was similar for the two age groups. Another limitation of the study is the limited amount of samples collected from each individual enrolled. Since the participants were young children and the amounts of stool collected were very limited, this did not allow us to retrieve the original samples that have discordant results to have further investigation. Additionally, we only used alcohol shocked culture enrichment on samples with discordant results between the GPP and DFS RT-PCR. It is plausible that some stools contained toxigenic *C. difficile* but tested negative by GPP and in-house RT-PCR assays because of low bacterial load, and culture enrichment is required to detect such cases, thus the calculated sensitivity of the two assays might have been inflated. Furthermore, the majority of our samples tested negative, and only 2.8% were positive when retested after alcohol shock and culture enrichment. This low positivity rate for *tcdB* may have skewed our percent agreement calculations. There is also potential for sampling error in our study due to the specific aliquot of stool chosen from each sample and the low volume of sample (200 μL of a 1/10 dilution of stool sample and eluted with 70 μL buffer) and 5 μL of extracted DNA was used for both GPP and DFS RT-PCR assays. While other studies have compared GPP to other methods of detection for *C. difficile* and other pathogens in several populations [8,10,11,14,15], this study is unique because it included the use of PCR following an enriched culture method to characterize the difference in performance between the two assays on stools collected from a pediatric population.

## 5. Conclusions

In conclusion, the Luminex xTAG^®^ GPP and our DFS in-house RT-PCR had high agreements for the detection of *C. difficile* toxin gene, *tcdB* with a PPA of 97% and a NPA of 99%. Multianalyte panels such as the Luminex xTAG^®^ GPP are high volume testing platforms with rapid turnaround times, no special setup, and are less labour intensive while providing identification of multiple different enteric pathogens [7,8]. This assay can easily be implemented in frontline microbiology laboratories and provides a rapid tool in making a diagnosis for enteric pathogens including *C. difficile*. However, our in-house DFS RT-PCR assay can also be used to detect toxigenic *C. difficile*, which is especially useful in a research setting as it is both sensitive and economical compared to the multianalyte panels or commercial assays available.

## Figures and Tables

**Table 1:** Primer and probe sequences used in this study [19].

Target, Primer/Probe	Sequence 5′ → 3′
*tcdB*-F	TACAAACAGGTGTATTTAGTACAGAAGATGGA
*tcdB*-R	CACCTATTTGATTTAGMCCTTTAAAAGC
*tcdB*-P	/56-FAM/TTTKCCAGT/ZEN/AAAATCAATTGCTTC/3IABkFQ/

**Table 2:** Discordant results for *tcdB* between the GPP and DFS RT-PCR resolved by PE RT-PCR.

GPP	DFS RT-PCR	PE RT-PCR	No. Samples (*n* = 15)
Positive	Negative	Positive	12
Negative	Positive	Positive	2
Positive	Negative	Negative	1

PE RT-PCR refers to results for *tcdB* after culture enrichment.

## Data Availability

Not applicable.

## Supplementary Materials

The following supporting information can be downloaded at: https://www.mdpi.com/article/10.3390/microorganisms10112214/s1, Table S1: Specificity panel for real-time PCR; Table S2: Sensitivity panel for determining the limit of detection of *tcdB* RT-PCR; Table S3: Ct values for *tcdB* from in-house RT-PCR for GPP/ DFS RT-PCR discordant samples.
