# Supplementary material for: Comparative Evaluation of Luminex xTAG® Gastrointestinal Pathogen Panel and Direct-From-Stool Real-Time PCR for Detection of C. difficile Toxin tcdB in Stool Samples from a Pediatric Population
Source: Microorganisms. 2022 Nov 9;10(11):2214. doi: 10.3390/microorganisms10112214 (PMC9693576; doi:10.3390/microorganisms10112214)
Supplement: Supplementary file 1 [file microorganisms-10-02214-s001.zip › microorganisms-1992155-supplementary.pdf]

Supplementary Materials:

Supplementary Table S1. Specificity panel for real-time PCR.

**Organism**	***tcdB***
Clostridioides difficile	+
Clostridium bifermentans, innocuum, perfringens, ramosum, septicum, sordellii, sporogenes, tertium, tetani	-
Aeromonas hydrophila	-
Bacteroides fragilities, vulgatus	-
Campylobacter coli, concisus, fetus, helveticus, hyointestinalis, jejuni, lari, upsaliensis	-
Citrobacter freundii	-
Edwardsiella tarda	-
Enterobacter aerogenes, cloacae, facecalis, faceium	-
Escherichia coli O103:H2, O111:H8, O121:H19, O145:NM, O153:H25, O157:H7, O165:H25, O174:H2, O26:H11, O45:H2, O8:H14	-
Klebsiella penumoniae	-
Micrococcus luteus	-
Morganella morganii	-
Non-toxigenic *C. difficile* stools (*n* = 14)	-
Plesiomonas shigelloides	-
Prevotella melaninogenica	-
Proteus mirabilis, vulgaris	-
Pseudomonas aeruginosa	-
Salmonella enterica ser. Braenderup, Brandenburg, Hadar, Heidelberg, Infantis, Javiana, Mbandaka, Montevideo, Newport, Oranienburg, Panama, Paratyphi A, Paratyphi B, Paratyphi B var. Java, Reading, Saintpaul, Schwarzengrund, Stanley, Thompson, Typhi, Virchow, Typhimurium	-
Serratia marcescens	-
Shigella boydii, Shigella dysenteriae, Shigella sonnei	-
Staphylococcus aureus, Staphylococcus epidermidis, Staphylococcus saprophyticus	-
Vibrio parahaemolyticus	-
Yersinia enterocolitica	-

Supplementary Table S2. Sensitivity panel for determining the limit of detection of *tcdB* RT-PCR. Dilutions of bacterial cell culture and the corresponding Ct values generated using our in-house RT-PCR assay. The PCR was performed in triplicates in 10 different runs on separate days, and mean Ct values were shown in Table below.

Colony Forming Units (CFU)/mL	Mean Ct Value	% Positive*
10^8	20.84	100%
10^7	24.57	100%
10^6	28.1	100%
10^5	31.59	100%
10^4	34.99	100%
10^3	37.86	87%
10^2	38.9	17%
10^1	Undetermined	NA

*% Positive indicates the percent positivity over 10 runs with triplicates in each run.

NA, not applicable

Supplementary Table S3. Ct values for *tcdB* from in-house RT-PCR for GPP/ DFS RT-PCR discordant samples (n = 15). Ct values above 35 were considered negative for *tcdB*.

PE, post enrichment

Sample	DFS RT-PCR *tcdB* Ct Value	PE RT-PCR *tcdB* Ct Value
1[#]	35.76	23.88
2[♦]	24.96	27.11
3[#]	35.02	24.06
4[#]	37.25	27.56
5[#]	38.09	24.62
6[#]	36.82	27.30
7[#]	37.09	24.18
8[#]	37.95	25.65
9[♦]	24.62	23.95
10[#]	36.14	23.00
11[#]	35.84	24.70
12[#]	39.27	27.54
13[#]	36.85	25.65
14[#]	> 40	24.71
15[#]	> 40	> 40

[♦]GPP negative/ DFS RT-PCR positive samples.

[#]GPP positive/ DFS RT-PCR negative samples.
